# Small airway impairment and bronchial hyperreactivity in patients with house dust mites–allergic rhinitis

**DOI:** 10.5415/apallergy.0000000000000193

**Published:** 2025-03-17

**Authors:** Yudan Wu, Hanxi Wu, Li Yao, Jiayi Zhu, Ailin Tao, Linmei Li

**Affiliations:** 1 The Second Affiliated Hospital, Guangdong Provincial Key Laboratory of Allergy & Immunology, The State Key Laboratory of Respiratory Disease, Guangzhou Medical University, Guangzhou, China

**Keywords:** Bronchial hyperreactivity, HDM-allergic rhinitis, IgE, small airway impairment

## Abstract

**Background:**

Patients with allergic rhinitis (AR) and bronchial hyperreactivity (BHR) may be at higher risk of developing asthma.

**Objective:**

The study was to investigate whether reactivity to house dust mites (HDM) and serum IgE level can be used to predict BHR.

**Methods:**

A total of 75 asthmatic patients were included in the study. All patients performed spirometry and underwent a bronchial challenge with histamine.

**Results:**

Seventy-five patients with HDM-positive skin prick tests who underwent airway provocation tests were included in this study. Small airway dysfunction was found in 19 patients. After the histamine challenge, the change of forced expiratory volume in the first second (FEV1) was more than 15% in 48 patients and more than 20% in 42 patients. There were positive associations between serum IgE levels and FEV1 fall value. The following cutoff values showed the best combination of sensitivity and specificity for AR with BHR: Serum total IgE 104.2 IU/mL (area under the curve [AUC]: 0.711, *P* = 0.0019), *Dermatophagoides pteronyssinus*–specific IgE 13.7 IU/mL (AUC: 0.799, *P* < 0.0001), *Dermatophagoides farinae*–specific IgE 27.8 IU/mL (AUC: 0.743, *P* = 0.0009).

**Conclusion:**

AR patients with HDM allergy have small airway dysfunction and airway hyperresponsiveness, and airway hyperresponsiveness is related to the degree of allergy. Simple measurement of allergen-specific IgE may be the best predictor of HDM-induced asthma in patients with AR.

## 1. Introduction

Allergic rhinitis (AR) is a common ailment with increasing prevalence in people of all ages, peaking in children and adolescents [1]. Triggers may include dust mites, airborne pollens, molds, and animals. House dust mites (HDM) are the most prevalent allergens in patients with rhinitis in China [2] and have been considered one of the most important triggers of asthma in children, adolescents, and adults [3]. Skin and blood allergy testing can be a useful diagnostic modality that may guide therapy. With the prolongation of the disease course, patients with AR may develop allergic asthma. Previous studies have shown that 15% to 38% of patients with AR have asthma, while 6% to 85% of patients with asthma also have rhinitis symptoms [4]. Similarly, many patients with localized AR report bronchial symptoms. Recent follow-up studies showed that the occurrence of symptoms suggestive of asthma increased from 18.4% to 24.4% in the first 5 years and to 30.7% after 10 years [5].

The forced respiratory flow rate at the 25% and 75% of the pulmonary volume (forced expiratory flow [FEF] 25-75) might be considered as a measure of the caliber concerning distal airways and as an early marker of small airway impairment [6]. Recent studies have shown that small airway disease is associated with more severe bronchial hyperreactivity (BHR), poorer asthma control, and more acute attacks [7]. Several cross-sectional studies also support the presence of small airway dysfunction in nonasthmatic children with AR [8, 9]. Nevertheless, no study data have explored the relationship between atopy parameters and small airway damage.

BHR is a key pathophysiological characteristic of bronchial asthma, marked by an increased airway responsiveness to nonspecific stimuli. This heightened reactivity results in smooth muscle contraction, elevated airway resistance, and airflow limitation, all of which are essential features of asthma. BHR is not only associated with the development of asthma but may also be observed in AR [10, 11]. BHR and allergic diseases in childhood will significantly increase the risk of asthma in adulthood [12]. These results suggest that BHR may be a preclinical state or an early pathophysiological change in the development of asthma in AR patients. In epidemiological studies, bronchial hypersensitivity is typically associated with atopy. There is evidence that not only the presence but also the degree of atopy is crucial in BHR to methacholine or adenosine monophosphate in patients with asthma [13].

The large number of AR patients, limited health resources, and the time-consuming and risk of bronchial provocation test (BPT) make it impossible to perform lung function detection and BPT for all AR patients to determine whether they are complicated with BHR. Therefore, exploring the risk factors of AR combined with BHR and conducting BHR detection in high-risk patients may also have health-economic significance. Thus, the aim of our study was to analyze how HDM sensitization patterns affect BHR and spirometric impairment in AR patients in South China.

## 2. Methods

### 2.1. Study population

The study retrospectively analyzed patients hospitalized for undergoing the BPT at the Center for Allergy of the Second Affiliated Hospital of Guangzhou Medical University between September 2019 and February 2023. A total of 75 patients with AR were enrolled in this study. The diagnosis of AR was in accordance with the diagnostic criteria of AR in the “Guidelines for the Diagnosis and Treatment of Allergic Rhinitis (2015, Tianjin).” All patients had atopy defined by a positive reaction to skin prick test (SPT) with HDM. The patient information is delineated within the table appended to the Supplementary Material 1, http://links.lww.com/PA9/A61. Enrolled patients had no prior history of asthma or other related respiratory conditions and exhibited no significant abnormalities on pulmonary imaging (X-ray or computed tomography). Additionally, patients were able to comply with the requirements for pulmonary function testing.

Patients were excluded from the study if they met any of the following criteria: (1) Systemic or topical use of hormones and other anti-infective drugs within 6 weeks. (2) Respiratory tract infection within 4 weeks. (3) Inhaled short-acting bronchodilators within 12 hours, oral short-acting β2 receptor agonists or long-acting β2 receptor agonists within 24 hours, and antihistamines within 48 hours. (4) Drinking alcohol or caffeine-containing beverages within 4 hours. (5) Unable to cooperate with pulmonary function testing.

The study was approved by the Ethics Committee of the Second Affiliated Hospital of Guangzhou Medical University (No.2020-hs-31, approved by Dr. Shikun Qian, the President of the Ethics Committee of the Second Affiliated Hospital of Guangzhou Medical University on August 2020). All patients provided written informed consent.

### 2.2. Detection of total/specific IgE levels in serum

Total IgE (tIgE) and specific IgE (sIgE) to *Dermatophagoides pteronyssinus* and *Dermatophagoides farinae* were detected by UniCAP100 automatic in vitro allergen diagnostic instrument of Pharmacia, Sweden.

### 2.3. Assessments and spirometry

Baseline spirometry was performed using a spirometer (Master Screen, JAEGER, Germany), and the following spirometry parameters were recorded as percentages of predicted values: (1)forced vital capacity (FVC), (2)forced expiratory volume in the first second (FEV1), (3)maximal mid-expiratory flow (MMEF), (4)maximal mid-expiratory flow after 50% of forced vital capacity has been not exhaled (MEF50), (5)maximal mid-expiratory flow after 25% of forced vital capacity has been not exhaled (MEF25).

### 2.4. Histamine bronchial challenge

Histamine bronchial challenge was performed to evaluate BHR only if basal FEV1 ≥70% of that predicted. Subjects inhaled increasing doses of histamine, starting from 0.07 mg. The scheduled doses consisted of the following: 0.07, 0.205, 0.825, and 1.1 mg. The test was interrupted if FEV1 was reduced by ≥20% of control or a maximal cumulative dose of 2.2 mg was achieved.

### 2.5. Degree of BHR

The positive rate of BHR was determined by the rate of decline in FEV1 after inhalation of the provocation, with less than 15% as negative, 20% or greater as positive, and 15% to 19% as suspicious positive.

### 2.6. Statistical analysis

All statistical analyses were analyzed using Graphpad Prism 9 (GraphPad, San Diego, CA, USA). Data were presented as mean ± SD, frequencies, and percentages, as appropriate. Correlations between variables were analyzed using the Spearman rank correlation coefficient. Correlation between tests was assessed by constructing a receiver operating characteristic (ROC) curve. A *P* value <0.05 was considered statistically significant.

## 3. Results

Patients’ characteristics are summarized in Table 1. A total of 75 patients (mean age 22.90 ± 12.43 years) were included in the analysis, comprising 44 males and 31 females. All rhinitis had a positive skin prick test response to HDM (2 positive, 13.33%, 3 positive, 25.33%, and 4 positive, 61.33%). Most patients showed positive sensitization to *D. pteronyssinus* (60, 80.00%) and *D. farinae* (63, 84.00%).

**Table 1.** The clinical characteristics of all patients

Demographic parameters	AR (n = 75)
Age, y	22.90 ± 12.43
Male/female	44/31
Serum total IgE, IU/mL	323.83 ± 308.93
Skin prick test, class	
2 positive	10 (13.33%)
3 positive	19 (25.33%)
4 positive	46 (61.33%)
*Dermatophagoides pteronyssinus*	62 (82.67%)
Number of positive	60 (80.00%)
Serum-specific IgE, IU/mL	33.47 ± 35.84
Class	3.63 ± 1.41
*Dermatophagoides farinae*	66 (88.00%)
Number of positive	63 (84.00%)
Serum-specific IgE, IU/mL	37.61 ± 35.06
Class	3.81 ± 1.45
FEV1% predicted	96.16 ± 11.41
FVC% predicted	98.13 ± 12.83
FEV1/FVC (%)	83.30 ± 7.05
MMEF% predicted	78.93 ± 20.09
MEF50% predicted	83.20 ± 21.8
MEF25% predicted	73.04 ± 24.66

AR, allergic rhinitis; FEV1, forced expiratory volume in the first second; FVC, forced vital capacity; MEF25, maximal mid-expiratory flow after 25% of forced vital capacity has been not exhaled; MEF50, maximal mid-expiratory flow after 50% of forced vital capacity has been not exhaled; MMEF, maximal mid-expiratory flow.

To further evaluate the association between the degree of atopy and lung function, subjects were stratified according to each atopy parameter (serum tIgE levels, *D. pteronyssinus* sIgE, and *D. farinae* sIgE, and number of positive SPT), spirometry indicates (FVC, FEV1, FEV1/FVC, MMEF, MEF50, and MEF25), and the value of FEV1 fall (Table 2). Significant positive correlations were observed for FEV1 fall% predicted and serum tIgE (*r* = 0.298, *P* < 0.01). We also found a significant correlation between the value of FEV1 fall and serum sIgE (*r* = 0.502 and *r* = 0.500, respectively) in patients with AR (*P* < 0.001). The number of positive SPT had a significant positive correlation with the value of FEV1 fall (*r* = 0.255, *P* < 0.05).

**Table 2.** Relationship between atopy parameters and spirometry

Demographic parameters	tIgE (n = 75)	*Dermatophagoides pteronyssinus–*specific IgE (n = 62)	*Dermatophagoides farina–*specific IgE (n = 66)	Number of positive SPT
FEV1% predicted	−0.101	−0.025	−0.025	−0.047
FVC% predicted	−0.120	−0.009	0.064	−0.132
FEV1/FVC (%)	0.008	−0.075	−0.083	−0.101
MMEF% predicted	−0.090	−0.120	−0.054	−0.068
FEF50% predicted	−0.131	−0.157	−0.084	−0.073
FEF25% predicted	−0.126	−0.047	−0.050	−0.012
FEV1 fall%	0.298*	0.502†	0.500†	0.255‡

FEF, forced expiratory flow; FEV1, forced expiratory volume in the first second; FVC, forced vital capacity; MMEF, maximal mid-expiratory flow; SPT, skin prick test; tIgE, Total IgE.

* *P* < 0.01.

† *P* < 0.001.

‡ *P* < 0.05.

Small airway dysfunction is the early expression of airway obstruction. FVC, FEV1, and FEV1/FVC are still in the normal range, but MMEF, FEF50%, and FEF75% can be significantly decreased. Table 3 reflects small airway function. Patients showed small airway dysfunction (<65%) in MMEF% predicted (18, 24.00%), MEF50% predicted (17, 22.67%), and MEF25% predicted (27, 36.00%). When 2 of the 3 indicators were lower than 65%, it could be judged as small airway dysfunction. The results in Table 2 show that 2 of the 3 indicators of 19 patients are below 65%, and 16 patients have all 3 indicators below 65%. These results suggest that small airway dysfunction exists in patients with AR allergic to dust mites.

**Table 3.** Baseline spirometry of small airways in patients with allergic rhinitis

Demographic parameters	AR (n = 75)
MMEF% predicted (<65%)	18 (24.00%)
MEF50% predicted (<65%)	17 (22.67%)
MEF25% predicted (<65%)	27 (36.00%)
2 of 3 indicators were lower than 65%	19 (25.33%)
All 3 indicators were lower than 65%	16 (21.33%)

AR, allergic rhinitis; MEF25, maximal mid-expiratory flow after 25% of forced vital capacity has been not exhaled; MEF50, maximal mid-expiratory flow after 50% of forced vital capacity has been not exhaled; MMEF, maximal mid-expiratory flow.

Responses to a bronchial challenge test with histamine in AR patients are shown in Table 5. About 42 patients (56%) showed a positive histamine challenge. Six patients had a FEV1 fall% between 15 and 20. There were no differences in FEV1% predicted and FVC% predicted between BHR-positive and BHR-negative patients. Additional lung function parameters decreased in the BHR-positive group, with the small airway function parameters reducing significantly. The BHR-positive group showed significantly higher tIgE and HDM sIgE. Patients with BHR had significantly higher numbers of positive SPT responses than those in the BHR-negative group (Table 4).

**Table 4.** BHR degree of patients with allergic rhinitis

Demographic parameters	AR (n = 75)
Positive (FEV1 fall% ≥ 20)	42 (56%)
Suspected positive (15 ≤ FEV1 fall% < 20)	6 (8%)
Negative (FEV1 fall% < 15)	27 (36%)

AR, allergic rhinitis; BHR, bronchial hyperreactivity; FEV1, forced expiratory volume in the first second.

**Table 5.** Results of ROC analysis of FEV1 fall level and atopy parameters

Parameters	tIgE (IU/mL)	*Dermatophagoides pteronyssinus–*specific IgE	*Dermatophagoides farinae–*specific IgE
FEV1 fall ≥15% vs FEV1 fall <15%			
AUC (95% CI)	0.711	0.799	0.743
Cutoff	104.2	13.7	27.8
Sensitivity	0.519/0.929	0.783/0.744	0.76/0.667
*P* value	0.0019	<0.0001	0.0009

AUC, area under the curve; CI, confidence interval; FEV1, forced expiratory volume in the first second; ROC, receiver operating characteristic; tIgE, Total IgE.

**Figure 1.**
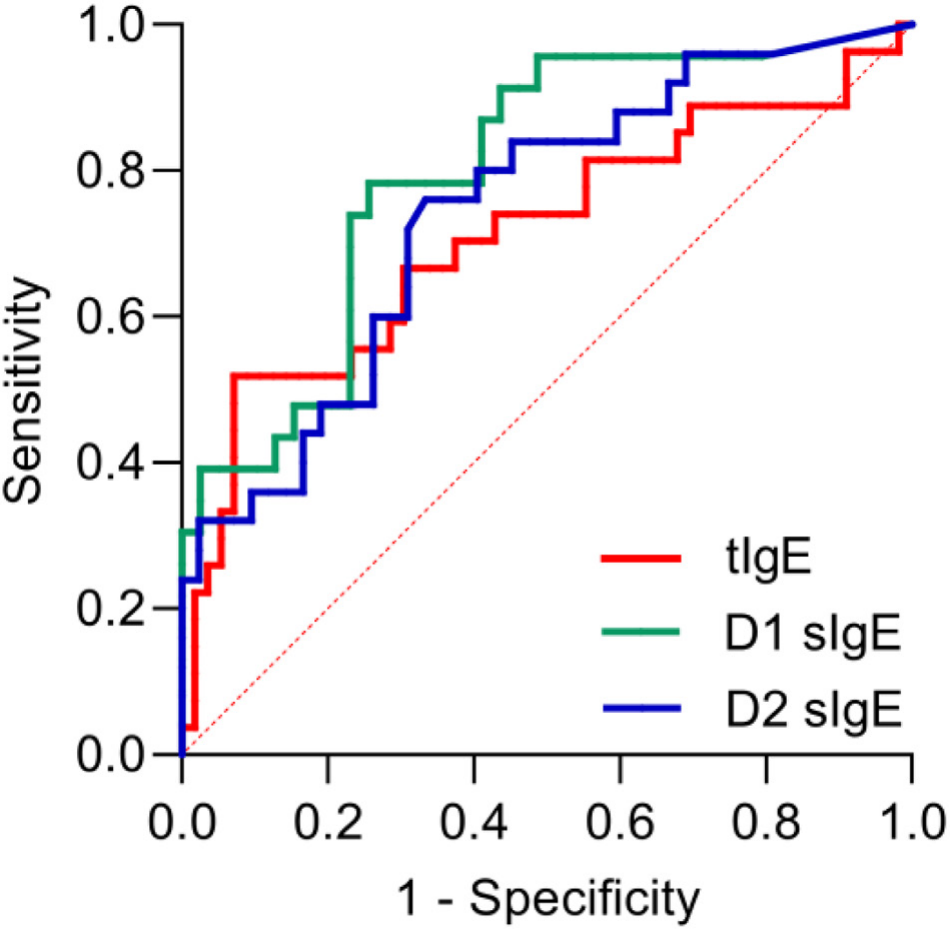
ROC curve analysis of tIgE, D1 sIgE, and D2 sIgE for BHR. BHR, bronchial hyperreactivity; ROC, receiver operating characteristic; sIgE, specific IgE; tIgE, Total IgE.

Significant positive correlations were observed for serum tIgE, D1 sIgE, D2 sIgE, and FEV1 fall values (Table 2). Serum tIgE, D1 sIgE, and D2 sIgE as predictors to identify BHR-positive patients were analyzed using the ROC curve (Figure 1). As shown in Table 5, the optimum cutoff point for tIgE was 104.2 IU/mL with an area under the curve (AUC) of 0.711 (*P* = 0.0019). In addition, the cutoff value of *D. pteronyssinus* sIgE was 13.7 IU/mL with an AUC of 0.799 (*P* < 0.0001). The cutoff value of *D. farinae* sIgE was 27.8 IU/mL with an AUC of 0.743 (*P* = 0.0009).

## 4. Discussion

AR is one of the independent risk factors for allergic asthma. Some patients with AR may have pulmonary dysfunction and airway hyperresponsiveness without asthma symptoms. BHR may be a preclinical state or an early pathophysiological change in the development of asthma in AR patients. However, BHR without typical asthma symptoms is often overlooked. The retrospective study confirms that AR patients have small airway dysfunction and a positive BHR response and that the degree of allergy is positively correlated with BHR.

Allergens are one of the key factors causing AR and positive bronchial provocation. Previous cross-sectional studies in children and adults have found that indoor perennial allergen sensitization is associated with the formation of BHR. Several studies have confirmed that HDM sensitization is a risk factor for AR with BHR. HDM allergen is one of the most important allergens in southern China. Therefore, the aim of this study is to evaluate the risk of BHR in patients with HDM-AR and explore the cutoff value of IgE for BPT.

The FEF25-75 might be considered as a measure of the caliber concerning distal airways and as an early marker of small airway impairment. Small airway dysfunction in the absence of asthmatic symptoms is a known feature of AR. For example, Ciprandi et al. [14] showed that 48% of patients with perennial AR alone showed reduced FEF25-75 values. Thayyezhuth et al. [15] confirmed that 5% of patients showed impaired post bronchodilator FEF25-75, which indicates the presence of small airway disease. Similar to findings from previous studies, our results demonstrated a prevalence of approximately 25.33% for small airway dysfunction in patients with AR. Therefore, spirometry should be considered in the long-term examination of patients with AR, as impaired FEF25-75 may indicate initial progression to asthma.

AR may contribute to worsening asthma through different pathophysiologic mechanisms: postnasal drip, nasal-bronchial reflex, oral breathing, and systemic allergic inflammation. In these subjects with normal FEV1 values, BHR may be envisaged as a marker of susceptibility to develop asthma. The study conducted by Helena et al. [9] on a cohort of 4,850 children revealed that patients sensitized to HDM were more prone to experiencing persistent and severe BHR compared with nonsensitized patients. One study included 365 AR patients residing in temperate continental regions, of whom 114 exhibited BHR [16]. In our study, the positive rate of airway hyperresponsiveness in patients with HDM-induced AR was as high as 56% to 64%.

IgE is elevated in allergic sensitization and is a common diagnostic marker for screening for atopic diseases [17]. Previous evidence has shown that high levels of IgE are associated with increased asthma prevalence, increased airway hyperresponsiveness, accelerated declines in lung function, and asthma control status [16, 18, 19]. A study by Buslau et al. [20] investigated the predictors of grass pollen allergy, and the total IgE of 95.5 kU/L was associated with AR and comorbid asthma. Borish et al. [21] found IgE levels of 124.3 kU/L in patients with childhood-onset asthma and 65.7 kU/L in those with adult-onset asthma. This finding is similar to our result with an optimal cutoff point of 104.2 kU/L (AUC: 0.82).

HDM sensitization has been reported in 50% to 85% of patients with AR and allergic asthma and plays a major role in driving airway disease development [22, 23]. Its presence in AR patients is a risk factor for the development of allergic asthma and poor asthma control in the future [24]. Schulze et al. [25] validated the predictors for early allergic asthma and determined that the optimal cutoff value for *D. farinae* sIgE was 19.6 kU/L (AUC: 0.88). Our findings also demonstrated that the optimal cutoff value for *D. farinae* sIgE was 27.8 IU/mL (AUC: 0.743, 95% confidence interval [CI]) and the cutoff value of *D. pteronyssinus* sIgE was 13.7 IU/mL (AUC: 0.799, 95% CI). The correlation and ROC analyses demonstrate a positive and significant relationship for serum sIgE and BHR to predict asthma.

In conclusion, this study suggests that some patients with AR have concomitant BHR, which is highly correlated with serum sIgE levels. Serum sIgE may be a candidate predictor of allergen-induced asthma in AR patients. Doctors should improve their understanding and strengthen the monitoring of IgE in AR patients. Only by early detection of AR patients with BHR and early intervention can the incidence of bronchial asthma be reduced.

## 5. Limitation of the study

In our study, which encompassed 75 patients diagnosed with AR and no history of asthma, we observed a significant correlation between serum levels of specific dust mite IgE and airway hyperresponsiveness. Consequently, we propose that serum dust mite sIgE may serve as a potential predictor for the progression from AR to asthma. However, this study has several limitations, including a relatively small sample size, a wide age range among participants, and incomplete detection results for sIgE levels in some patients. Additionally, while statistical significance does not necessarily equate to clinical significance, the observed association warrants further consideration.

## Acknowledgements

This work was supported by National Science and Technology Innovation 2030 Major Project (2023ZD0406303), Tertiary Education Scientific research project of Guangzhou Municipal Education Bureau (202235411), and National Natural Science Foundation of China (81871266).

## Conflicts of interest

The authors have no conflicts of interest.

## Author contributions

The study conception and design: Yudan Wu, Hanxi Wu, Li Yao, Jiayi Zhu, Ailin Tao, and Linmei Li. Acquisition of data: Yudan Wu and Linmei Li. Analysis and interpretation of the data: Yudan Wu and Hanxi Wu. Statistical expertise: Li Yao and Jiayi Zhu. Critical revision of the article for important intellectual content: Ailin Tao and Linmei Li. Final approval of the article: Yudan Wu, Hanxi Wu, Li Yao, Jiayi Zhu, Ailin Tao, and Linmei Li.

## Supplementary material

Supplementary Material 1 can be found via 10.5415/apallergy.2022.12.e38

Supplementary Material 1

Click here to view
